# Harnessing Artificial Intelligence, Machine Learning and Deep Learning for Sustainable Forestry Management and Conservation: Transformative Potential and Future Perspectives

**DOI:** 10.3390/plants14070998

**Published:** 2025-03-22

**Authors:** Taojing Wang, Yinyue Zuo, Teja Manda, Delight Hwarari, Liming Yang

**Affiliations:** State Key Laboratory of Tree Genetics and Breeding, College of Life Sciences, Nanjing Forestry University, Nanjing 213007, China; wangtaojing@njfu.edu.cn (T.W.); 18305525894@163.com (Y.Z.); teja.manda27@gmail.com (T.M.)

**Keywords:** sustainable forest management, artificial intelligence, machine learning, deep learning, forest conservation

## Abstract

Plants serve as the basis for ecosystems and provide a wide range of essential ecological, environmental, and economic benefits. However, forest plants and other forest systems are constantly threatened by degradation and extinction, mainly due to misuse and exhaustion. Therefore, sustainable forest management (SFM) is paramount, especially in the wake of global climate change and other challenges. SFM ensures the continued provision of plants and forests to both the present and future generations. In practice, SFM faces challenges in balancing the use and conservation of forests. This review discusses the transformative potential of artificial intelligence (AI), machine learning, and deep learning (DL) technologies in sustainable forest management. It summarizes current research and technological improvements implemented in sustainable forest management using AI, discussing their applications, such as predictive analytics and modeling techniques that enable accurate forecasting of forest dynamics in carbon sequestration, species distribution, and ecosystem conditions. Additionally, it explores how AI-powered decision support systems facilitate forest adaptive management strategies by integrating real-time data in the form of images or videos. The review manuscript also highlights limitations incurred by AI, ML, and DL in combating challenges in sustainable forest management, providing acceptable solutions to these problems. It concludes by providing future perspectives and the immense potential of AI, ML, and DL in modernizing SFM. Nonetheless, a great deal of research has already shed much light on this topic, this review bridges the knowledge gap.

## 1. Introduction

Plants are important elements of the ecosystems, facilitating carbon exchange and supplying food and habitats for both animals and humans. In terms of their diversity and population, plants from forests are on the verge of extinction due to overuse and mismanagement. Therefore, there is a need for urgent and conscientious stewardship of these natural reservoirs for continued provision and sustenance [1]. Sustainable forestry management (SFM) has become a central concern in today’s world, although limited by productivity rise, environmental degradation, and global climate change [2]. In detail, SFM refers to the conservative balancing of ecological, social, and economic objectives to guarantee the permanence and resilience of natural ecosystems, especially forest plants, while benefiting from them [3]. Despite the conservation efforts, forest plants’ survivability is under constant threat of deforestation and degradation. Factors such as agricultural extensions, logging, infrastructure development, and urban movements lead to plant depletion, animal habitat loss, biodiversity regression, and increased carbon emissions [4]. These factors are fueled by poor monitoring, weak governance and law enforcement, and insufficient resources, thus emasculating SFM. Weak law enforcement leads to conflicts over land use, including illegal mining and competition for forest resources among these issues [4,5]. In addition, climate change has increased challenges in forestry management through the increased incidence and intensity of extreme weather events, as well as the spread of plant pests and diseases [6,7]. These challenges in SFM affect plant species composition and compromise their survival [8].

Therefore, sustainable forestry management plays an essential role in environmental conservation and resource management; it ensures the protection and perpetuation of plant trees, wildlife niches, and other forest systems [9]. In addition, it provides ecological balance, ensures habitats for an array of species, both animal and plant, helps mitigate climate change, and aids plant populations and diversity [10,11]. In essence, sustainable forestry management is a cornerstone of broader environmental conservation efforts, as it helps maintain healthy ecosystems, supports biodiversity, and contributes to global efforts to address climate change.

On the other hand, the advent of artificial intelligence (AI) has modernized SFM by providing innovative solutions for monitoring, modeling, decision making, and law enforcement [12]. Artificial intelligence refers to a broad field of operation incorporating the development and capability of computer systems to perform tasks that otherwise require human intelligence [13]. Included in this broad set are deep learning (DL) and machine learning (ML); generally, these learning techniques use AI networks to learn and make predictions from the data provided [14]. Their generation is inspired by the structure and functions of the human brain, forming artificial neural networks (interconnected through nodes similar to the human brain). These networks automatically self-learn data presentations and progressively enhance their internal representations through a process called training [14]. For instance, AI algorithms merged with satellite images and remote sensing data can be used to automate the monitoring of plant growth, distribution, and population changes in forests. Techniques in DL, including convolutional neural networks (CNNs), can be trained to monitor and sense any changes in the forest plant cover, learn and identify plant tree species, analyze and record plant height and diameter, and assess plant health [15]. These capabilities are essential for SFM to balance economic objectives with environmental sustainability and ensure forests can continue to provide essential services for future generations. By integrating machine learning into forestry practices, we can enhance our ability to preserve and sustainably manage forest plants in the face of growing environmental pressures and help optimize forest management practices at both spatial and temporal scales [4,5,6,7]. Therefore, harnessing the power of AI, ML, and DL can improve conservation efforts, alleviate plant misuse and exhaustion, and promote SFM while benefiting the present and future generations.

## 2. Challenges in Sustainable Forestry Management

Sustainable forestry management (SFM) aims to provide balanced economic, ecological, and social goals, ensuring the long-term health and productivity of forest plants [16]. However, the implementation of SFM strategies is inhibited by several factors summarized as environmental, social, economic, institutional and government, technological, and region-specific challenges [16].

### 2.1. Environmental Challenges

**Figure 1:**
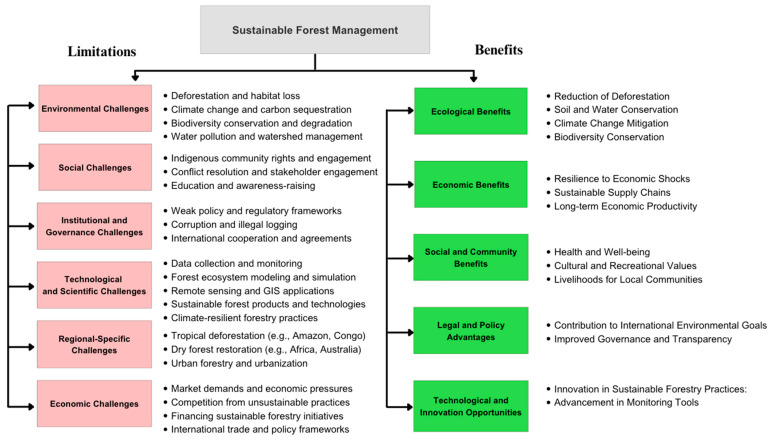
Benefits and limitations in sustainable forestry management.

The loss of forest plants due to deforestation, biodiversity loss, and climate change has increased the importance of SFM [17]. Despite rising concerns and sustainable practices, SFM is still impeded by several environmental challenges (Figure 1). Deforestation, one of the most significant challenges, refers to the large-scale removal of plants, including trees and other plants, from forests for timber, agriculture, and urban development [18]. Drivers of deforestation include human population increase, improper land use, industrialization, and poor forest management [19]. Importantly, deforestation ignites a succession of irreversible environmental changes that, to a greater extent, reduce the benefits drawn from forests [17]. Both of these factors are reflected by visible impacts such as soil erosion, siltation, fluctuations in weather patterns, and reduction in clean water access [20]. According to the FAO, the world loses approximately 10 million hectares of forest land annually [21]. Deforestation affects annual rainfall; a study demonstrated reduced precipitation over deforested regions across the tropics [22,23]. The effects of deforestation on precipitation increase at large scales greater than 50 km, and it is estimated that 1% of forest loss reduces precipitation by at most 0.5 mm per month [22,23]. Thus, SFM practices aim to balance logging with forest regeneration efforts, meeting global demands without over-exploitation. Plants are the main players, acting as carbon sinks and absorbing significant amounts of carbon from the atmosphere [24]. Deforestation and forest degradation release carbon back into the atmosphere, increasing the greenhouse effect and contributing to global warming, hence climate change [25]. In 2020, the Intergovernmental Panel on Climate Change, IPCC, estimated that deforestation accounts for almost 10% of global carbon emissions [26]. Additionally, climate change intensifies other environmental challenges to SFM. For instance, inconsistent weather patterns and shifting precipitation patterns as a result of climate change increase the vulnerability of forest ecosystems to wildfires, pests, diseases, cyclones, hurricanes, etc. SFM plays an essential role in adapting to these changes and implementing climate-resilient practices such as afforestation, reforestation, and other adaptive measures [25,26].

Like animals, plants pose a fundamental challenge to SFM (Figure 1), especially those species often introduced by human activity [27]. These tend to outplay native species for resources, disrupting ecosystems and damaging biodiversity. For example, in Europe and North America, invasive species have caused widespread plant tree mortality [28,29]. Human activities like logging and clear-cutting facilitate the spread of invasive species. When established, invasive species are difficult to control, making prevention a vital strategy in SFM. Thus, prevention is more difficult considering the increase in world trade and travel.

### 2.2. Social Challenges

Sustainable forest management is faced with countless social challenges that require imperative attention to guarantee the long-term productivity of forests [30]. Addressing these aspects requires a universal approach integrating the demands of forest-based communities, native rights, and ecological balances [31,32]. The most crucial social challenge is the issue of land tenure and property rights (Figure 1). In developed and developing countries, land ownership and user rights are unclear or contested. Natives and local communities can contest rights with the local government, and this anomaly often ignites conflicts that demoralize SFM [32]. For effective forestry management, SFM must include the participation of Indigenous people in decision making. This shows respect for their knowledge systems and recognition of their land rights. Nonetheless, achieving this level of participation is often impeded by historical discrepancies, power inequities, and differing perspectives on land use [33].

### 2.3. Economic Challenges

In most parts of the world, forests serve as a source of dependence for most rural and forest-dependent communities [34]. Plants are used for construction, food, landscaping, and other cultural purposes. These economic dependencies often lead to illegal logging, overharvesting, and illegal mining [34,35]. Thus, poverty limits the efforts of sustainable forest management (Figure 1). SFM should address the principal causes of poverty in these regions for effective sustainable forestry management. This involves creating employment, providing access to markets for sustainable products, and providing financial and technical support for sustainable practices [36]. However, it is almost impossible to alleviate poverty while promoting forest conservation. One way of fostering a certain degree of success in SFM is engaging local communities in decision-making processes. Engaging community participation presents several challenges, including a lack of trust between communities, the government, and all other parties included [31]. SFM should seek to first build trust, strengthen community institutions, and ensure that participatory processes are genuine [31].

Weak governance, corruption, and inadequate law enforcement of forestry laws contribute to the challenges of SFM [37]. For instance, in governments that fail to enforce regulations or allow illegal practices, sustainable forestry management practices are undermined, and local rights are violated. In most cases, governments may prioritize short-term economic benefits over long-term sustainability, leading to policies that favor industrial-scale logging or agricultural expansion over forest conservation [38,39]. Numerous economic challenges complicate the implementation of sustainable forestry management [40]. To overcome these challenges, it is imperative to create financial schemes that support sustainable practices and implement policies that align with environmental conservation [41]. Another key economic challenge in SFM is the high cost associated with sustainable practices. Almost all activities associated with sustainable forestry require intensive capital, time, and resources, labor [40]. For example, the regeneration of forests after logging demands concise planning, monitoring, and direct capital investments [41]. While sustainable practices aim to ensure long-term forest productivity, product pricing is often a barrier to sustainable management [42]. Sustainable forestry products often struggle to compete in the global market, where price is a dominant factor [42]. For instance, timber from sustainable forestry practices might generally be costly due to the high cost of maintenance and harvesting as compared to timber from unsustainable practices [43,44].

Also, competing demands for land limit the effectiveness of SFM; forest lands are alternatively utilized for agriculture, mining, and urban development [40]. Since these industries offer quick and greater financial returns, the opportunity cost of maintaining forests for sustainable practices can be expensive, especially in developing countries where economic growth is of prime importance [45]. In retrospect, industries such as agriculture, logging, and mining are often more profitable than sustainable forestry, especially in the short term [42]. Thus, these alternative industries present a huge challenge to sustainable forestry management, and this undercuts the feasibility of sustainable forestry, especially in weak and corrupt governance [42].

While sustainable forestry offers long-term benefits, many stakeholders lack adequate financial resources. This particularly impedes small-scale enterprises, indigenous communities, and smallholders [46]. Nonetheless, government incentives and financial programs provide financial assistance to smallholders in substantial forestry, although these services are not widely available to all.

## 3. AI, ML, and DL in Sustainable Forest Management

**Figure 2:**
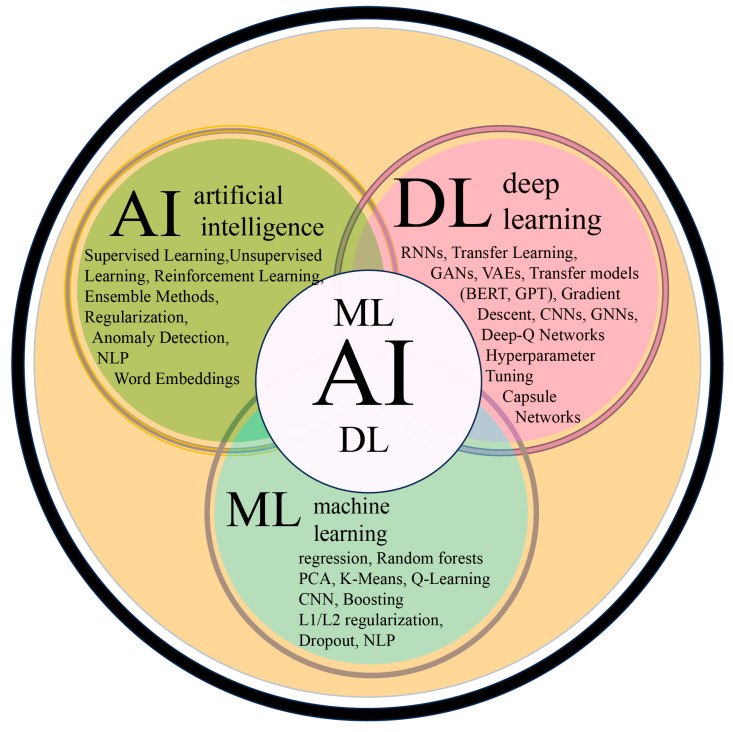
Illustration of the relation and differences among AI, ML, and DL. AI incorporates the broader concept of machines performing tasks that otherwise would be performed by human intelligence. ML, a subset of AI, uses algorithms that self-learn from input data. DL a further subset, makes use of multi-layered neural networks to enhance the accuracy of predictions and decision-making.

Forests are crucial components of the environment, essential for conservation. For ages, their management and protection have relied on traditional methods [47]. Nonetheless, the advent of artificial intelligence (AI), machine learning (ML), and deep learning (DL) have transformed forestry management [48]. Primarily, AI automates and enhances the analysis of complex datasets obtained from digital technologies, including satellites, drones, and aerial imagery, mimicking human intelligence [49]. AI processes and interprets vast amounts of data, reducing the reliance on manual labor (Figure 2). Machine Learning (ML), a subset of AI, works on algorithms that provide innovative solutions to compact environmental challenges through the efficient and precise analysis of vast amounts of data. ML facilitates better decision making and precise forest resource management [50]. Deep learning (DL), a further subset of ML, utilizes techniques that are an increasingly important feature in sustainable forest management (SFM), helping optimize forest resources more effectively [51]. DL systems utilize large-scale, high-dimensional data mined from satellites, environmental sensors, and other components (Figure 2) [52].

Artificial intelligence is progressively being incorporated into sustainable forestry management to address challenges related to conservation [53], resource management [54], and plant protection [55]. This has enabled forest management and aided in decision making, thereby improving sustainability. AI-powered tools obtain data mostly from remote sensors, where remote sensing entails the use of satellite imagery, aerial photographs, and the use of light detection and ranging (LiDAR) to provide a synoptic view.

**Figure 3:**
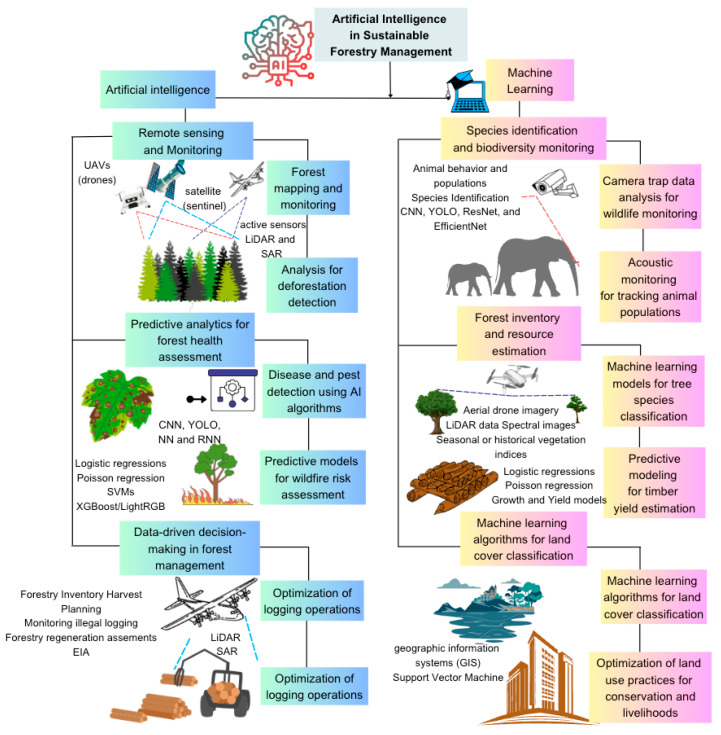
Overview of AI- and ML-based systems applied in sustainable forestry management. The image highlights critical applications, such as remote sensing for forest health monitoring, predictive modeling for timber production optimization, automated detection of unlawful logging, and climate impact assessment. These functions merge machine learning, computer vision, and data analytics to promote sustainable practices while promoting ecological and economic outcomes. In addition, they utilize several AI-driven algorithms, which are shown in the image with graphical representations.

Remote sensing systems are available as diverse arrays of sensors and platforms; sensors can be divided into passive and active sensors. Passive sensors detect natural radiation emitted or reflected by the scene or object under observation without emitting any energy [56]. Examples include visible light cameras or thermal infrared sensors. On the other hand, active sensors emit their energy to measure the portion of energy reflected or emitted back by an object or scene in the observation [56]. Examples include the radar and LiDAR systems that emit radio waves or laser pulses, respectively, and then analyze the return signal. Platforms used by AI in SFM range from satellites, planes, UAVs (unmanned aerial vehicles), and rotaries [57]. A commonly used AI-powered remote sensor in SFM is an optical image; it acquires data beyond visible wavelengths across the electromagnetic spectrum. Followed by LiDAR (light detecting and ranging) and SAR (synthetic aperture radar) active sensors (Figure 3). LiDAR data are presented by 3D point clouds; the data are then classified as categorical or continuous outputs. For instance, land use and cover can be classified categorically, while the projective vegetation cover is continuous. LiDAR capitalizes on the speed and accuracy of AI platforms to translate a high capacity and produce complete, accurate point clouds over large expands of forest cover.

ML techniques are classified as supervised or unsupervised techniques mainly based on the patterns in which they recognize and collect data [58]. The main difference is that, in the supervised class, algorithms are trained to employ labeled data to catalog pixels or objects as forest or non-forest. In unsupervised classification, data are clustered into groups centered on spectral properties and then categorized accordingly.

DL utilizes artificial neural networks to analyze data and improve forest-related decision making in SFM (Figure 3). Established DL algorithms, such as CNNs, automatically learn features (lazy learning) from captured data [59]. Thus, they efficiently and quickly process data from satellite imageries and climate data, monitor forest health, predict deforestation risks, optimize timber harvesting, and assess carbon sequestration [48].

AI, ML, and DL have already been applied in diverse applications, including forest cover measurements, vegetation structure analysis, moisture biodiversity, and soil characteristics.

## 4. Applications of AI in Sustainable Forestry Management

### 4.1. Forest Monitoring and Mapping

AI utilizes remote sensing systems in forestry management for monitoring and mapping. These systems work primarily through advanced capabilities to provide analytical data on forest cover, health, biomass, and any changes over time [55]. AI-based systems have been employed to combat illegal logging by analyzing satellite images and monitoring forest activities in real time (Figure 3). The systems can work as bioacoustics analysis to detect unusual noises, such as chainsaws, vehicles, or human voices, that may indicate illegal logging activities [60]. Organizations like Rainforest Connection (RFCx) use AI to analyze forest sounds captured by devices called “guardians” [61]. Recently, Hitachi Vantara collaborated with RFCx to develop AI algorithms that can predict logging events with 96% accuracy, giving rangers a 5-day advance notice [62]. Furthermore, AI coupled with Internet of Things (IoT) technology has enabled the creation of sensor networks that can monitor forests more effectively [62]. These systems can detect and report illegal activities in real time. Some initiatives repurpose old smartphones, equipping them with AI technology to create cost-effective monitoring devices for rainforests [63]. Companies like Timbeter use AI smartphones and portable devices to accurately measure and track timber, which helps in combating illegal logging by providing transparent and verifiable data on timber harvests [64]. By shifting from reactive to proactive measures, AI has significantly boosted efforts to protect forests and combat illegal logging. These AI-based approaches not only improve the detection of illegal logging but also enhance the safety of forest rangers by providing them with crucial information before they enter potentially dangerous situations.

Unmanned aerial vehicles (UAVs), commonly known as drones, have emerged as powerful tools for mapping and monitoring in various fields, including agriculture, environmental conservation, urban planning, and disaster management (Figure 3). Their increased usage largely depends on their ability to capture high-resolution imagery and generate detailed data over large areas [65]. This significant increase in drone usage highlights their importance in various fields, including forestry inspection and mapping [66]. In the context of mapping and tracking, AI contributes in several ways; for instance, in Autonomous Navigation, AI enables drones to navigate autonomously, avoiding obstacles and following predefined paths. This is crucial for accurate mapping and efficient monitoring of large and inaccessible areas. These UAVs can be flown along predetermined transects to collect images that are later processed into orthomosaics and digital surface models (DSMs), which are essential for creating accurate vegetation maps and estimating species coverage [67]. Moreover, the use of UAVs in forestry is not only for monitoring the condition of forests but also for predicting changes, which is crucial for conservation and protection measures [68]. Interestingly, while UAVs offer substantial benefits for forest monitoring, such as cost savings and minimal impact on sensitive habitats, they also present challenges, including regulatory compliance, data security, and technical limitations.

The increasing rate of global deforestation raises alarms of concern and significant threats to global ecosystems [17]. Elimination of the drivers and patterns of deforestation offers strategies to mitigate deforestation. Nonetheless, satellite imagery has emerged as a powerful tool for identifying and monitoring deforestation [69]. Therefore, mapping deforestation using satellite imagery provides significant insights into the spatial and temporal patterns of these issues; identifying hotspots and developments of targeted intervention can be easily imposed [70,71]. The fundamental principle of satellite imagery analysis in deforestation detection is embedded within satellite image comparison of the same region captured at different time intervals [72]. Completing this process includes two important stages: leveraging complex image processing techniques and machine learning algorithms [73]. By analyzing and superimposing captured images, changes can be made in land cover, mainly focusing on regions with replaced dense forest cover (Figure 3). Utilizing this method aids in the differentiation of deforestation from natural causes, such as seasonal vegetation and human causes [74]. During the satellite imagery process, satellites capture data across various wavelengths of the electromagnetic spectrum; healthy vegetations reflect a light type different from uncovered soils or recently cleared soils [75]. Additionally, analysts leverage spectral indices—like mathematical formulas including datasets from specific wavelengths. For example, the Normalized Difference Vegetation Index (NDVI) is used to highlight areas with high vegetation cover. Therefore, by analyzing changes in the spectral indices, deforestation events are identified.

ML offers exciting advantages; it offers trained algorithms on immense datasets of satellite images to automatically identify arrays related to forest clearing [76,77]. High-resolution satellite images can be obtained from various satellite sources, including Landsat, Sentinel, and others like DigitalGlobe (Figure 3). Research in southeast Mexico has used Landsat images and ML-based BFAST algorithms to analyze deforestation and land surface temperature changes [78]. Their comparisons against users and producers showed that deforestation maps from ML-based Landsat values were effective and were better tools for examining deforestation. Spatial, temporal, and spatiotemporal deep learning methods combined with satellite imageries over six years were used to effectively measure the rate of deforestation and predict future land uses and decision making in land use control [79]. Additional uses of ML in deforestation are shown in Table 1.

**Figure 4:**
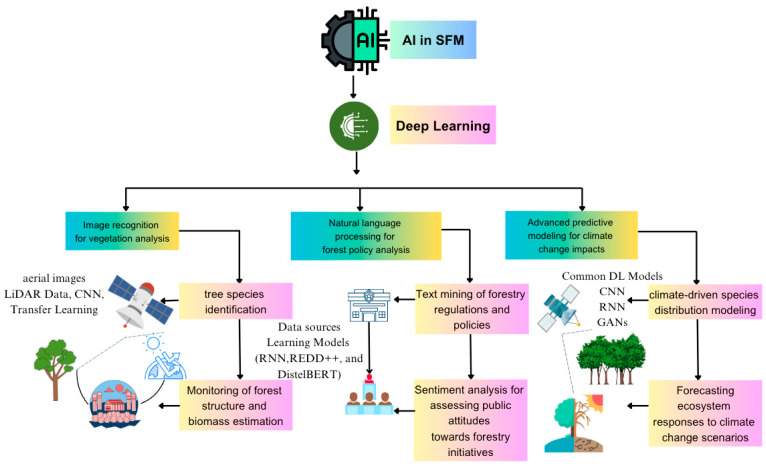
Roles of deep learning models in sustainable forestry management. The figure illustrates the use of deep learning in key areas including forest monitoring, resource evaluation, conservation of biodiversity, and climate change. Their application includes satellite imagery analysis for deforestation identification, species classification through automated sensors, and carbon stock evaluation for climate change impact assessment. Using DL algorithms and regression models to facilitate evidence-based decision making and sustainable practices in forestry.

Deep learning algorithms (DLAs) have been trained to assess tree characteristics and measure parameters easily for a decade. These include the branch, plant, leaf (photographic image), leaf (scan or scan-like image), flower, and stem. Transfer learning, AlexNet, VGGNet, and GoogLeNet are common DL models used to identify tree species based on LiDAR and drone-acquired images [94]. Even when distinguishing broad leaf and conifer species, though, traditional plant species are challenging and laborious, requiring great expertise [95]. CNNs are used in measuring plant leaf metrics using shallow recognition architecture and feature histograms of oriented gradients (HOGs) (Figure 4). Ogana et al. [48] trained CNN-DL models to predict tree heights in the rainforest of Nigeria. Additionally, the predicted tree heights were used as models to estimate aboveground biomass. CNNs (Faster R-CNN) and open-source aerial RGB imagery were utilized to detect temperate forests’ upper canopy layers [96,97]. This strategy accurately informs forest inventories and focuses on management measures to build resilient forests.

DLAs are also used to automatically map individual crowns of Vismia (low-resilience recovery indicator), Cecropia (fast recovery indicator), and trees in general. Additionally, they are used in geolocating individual trees and identifying their species in heterogeneous forests. For example, DLAs were used in the field screening of wood species in support of the Convention on International Trade in Endangered Species of Wild Fauna and Flora (CITES) [98]. Information on the spatial distribution of palm trees in tropical forests is crucial for management and commercial purposes. RGB images from UAVs, in combination with DL modes, CNN, and ResNet, help identify and geolocate palm trees in the Amazon, thereby supporting management projects and community-based monitoring programs [99]. In vineyards, variety selection monitoring and harvesting is often tedious due to large-scale operations and the number of species. However, transfer learning and other techniques based on a DL mode, AlexNet, have enabled the ease of variety identification, disease monitoring, and harvesting in vineyards [100]. Additional current applications of DLAs in SFM are listed in Table 1.

### 4.2. Predictive Analytics for Forest Health

The integration of AI models and predictive analysis provides real-time analysis and suggestions important for SFM [49]. Drone remote sensing applications combined with LiDAR technology in forest management are becoming increasingly appealing due to their low cost, high resolution, and ease of applicability. AI-powered drones can also be used to collect high-resolution data on forest characteristics and allow additional activities to enhance forest health and productivity [49]. Commonly used AI-automated models for 3D modeling tree information such as canopy, trunk, and height, include Tree Information modeling (TIM) and Forest Digital Twins (FDT) [101]. Also, Airborne Laser Scanning (ALS) and Terrestrial Laser Scanning (TLS) as subsets of point clouds are utilized to represent tree canopy fields [101]. Additionally, AI-based systems work in predictive modeling to analyze changes in the forest’s bioacoustics signatures—the normal collective sounds of animals in the forest (Figure 3) [102].

AI algorithms are revolutionizing disease and pest detection in agriculture, offering significant improvements in accuracy, speed, early identification and management, and efficiency compared to traditional methods (Figure 2; Table 1) [12]. These AI-driven systems utilize machine learning and computer vision to analyze data from various sources, such as sensors and images, and detect anomalies indicative of disease or pest presence, thereby facilitating timely interventions. ML-based systems have shown tremendous performance in the classification of plant diseases and pest images. For example, the VGG16 CNN merged with hyperspectral image processing techniques was used in the classification and diagnosis of citrus diseases and pests [103]. An improved version of VGG16, the VGG-INEP neural network has been used for its ability in multi-scale feature extraction. It has enhanced disease surveillance and detection. Combined with Rainbow, CGG-INCEP is used for early apple disease detection. In other studies, outputs of VGG16, SVM, and SoftMAX were used for citrus fruit disease classification [104]. ML models analyze historical data and other environmental variables to forecast potential outbreaks (Figure 3). Pérez-Romero et al. [105] used a combination of remotely sensed data and ML models to predict the spread of the pine processionary month. These play a crucial role in enhancing plant disease and pest detection, contributing to improved plant management and sustainability. Also, DL models have been trained from historical forest data to forecast future forest dynamics such as epidemiolocal prevention strategies [106]. In forestry, drones equipped with DL algorithms are used for wheel rut detection and post-harvest assessment [107]. Another deep learning-based image segmentation taken from UAVs has been used to predict *Thaumetopoea Pityccampa* Tents (TPTs) in aerial imagery. These geolocations of detected TPTs are further calculated and converted to spatiotemporal maps that use navigation logs and parameter UAVs [108]. Other DL models, such as recurrent neural networks (RNNS) are effective in handling time-series data, which is essential in disease prediction and spread over time. Kim et al. [109] used the long short-term memory recurrent neural networks (LSTM-RRNs) in the early forecasting of rice blast disease. Additional applications in predictive analytics for forest health are shown in Table 1.

### 4.3. Wildfire Detection and Management

AI-powered models are crucial in wildfire risk management and are effective in decision making and resource allocation. These models integrate static and dynamic components useful in forecasting wildfires; they implement spatial data and weather patterns to predict ignition sites (Figure 3) [110]. Recent research in forestry management has proposed a platform that uses constantly patrolling UAVs in potential fire areas and utilizes AI to recognize and detect smoke or fire based on the still images or videos captured by patrolling drone cameras [111].

Machine learning models have provided a significant advancement in wildfire prediction capabilities over traditional fire danger rating indices (Figure 3). ML models utilize remote sensing technology (such as satellites and UAVs) to focus on calibrating fuel moisture models within a fire danger rating system and improve fire management tools [112]. The forest fire detection algorithms enabled by ML techniques consist of rule-based color models that use both RGB and YCbr color spaces to identify fire pixels [113]. A study proposed a forest fire detection algorithm with great advantages for real-time forest fire detection. It exploits YOLOv3 on UAV-based aerial images; in this technique, a small scale of CNN is implemented with the aid of YOLOv3 after capturing images by the UAV platform (Figure 3) [114]. Also, Assilzadeh et al. [115] have formulated a framework that uses multi-sensor applications for monitoring fire danger fire activity linked with decision-aid models in a GIS environment. Similarly, LiDAR-derived applications work within the ML framework to detect and describe forest disturbances or changes, including fire extending [116]. These frameworks employ wildfire risk mitigation strategies and provide operational platforms that offer real-time fire danger forecasts, which are crucial for prevention and pre-suppression planning [117].

Other ML models emphasize the importance of understanding wildfire spread behavior and risk assessment for prevention and mitigation and propose a risk-science approach that integrates social, ecological, and fire management needs, which could be informed by predictive models [118]. For example, Rodell et al. [119] recently introduced an hourly Fire Weather Index system that uses ML models to improve traditional daily indices by capturing severe fire weather variations. Additional applications in wildfire detection and management are shown in Table 2.

### 4.4. Biodiversity Conservation

AI models analyze data from camera traps, satellite imagery, and historical data of species at risk of extinction. These models use environmental data to assess the ecological needs of future possible changes (Figure 3). Most recent AI tools, such as CAPTAIN, further quantify the trade-offs between costs, benefits, and biodiversity protection. This further allows the exploration of multiple biodiversity metrics [120]. Other tools, such as reinforcement learning (RL), genetic algorithms (GAs), and optimization algorithms (OAs), optimize designs to protect nature reserves by evaluating multiple factors, including biodiversity values, connectivity, and land use [121]. These tools assess which areas maximize conservation benefits costs–land acquisition and managing human–wildlife conflicts [122]. Recently, a study predicted the regional distribution of *Rhododendron arboretum*, a medicinal plant, using BIOCLIM (an advanced species distribution model) [123]. Input parameters were obtained from satellite missions, MODIS, Sentinel-2/5P, ECOSTRESS, and SRTM.

AI tools are also employed to optimize wildlife corridors, ensuring safe migration and protected habitats and maintaining genetic biodiversity. These systems utilize camera traps, satellite images, and drone footage data to identify suspicious behaviors or environmental changes indicative of human threats to forest conservation. A recent study has proposed autonomous surface vehicle (ASV) model systems for the surveillance of marine areas through AI-based image recognition. This technology uses the smart algorithm, SAAO, to detect illegal practices [124]. AI models are also used in monitoring ecosystems to detect the presence of invasive species that might be a threat to biodiversity (Figure 3). This is crucial in sustainable forest management since early detection of invasive species in sensitive ecosystems allows swift eradication and containment efforts. Recently, a study validated a workflow based on this technology using AI tools GoeSLAM scanner and VirtSilv to offer solutions and technical advantages for restoring biodiversity [125]. Another study used a low-cost RPA to monitor forest restoration (FR) quality through mapped key indicators using drones and AI in the Amazon [126].

Unlike AI, ML applications require modeling tools like deep neural networks, GIS, and CNNs to predict suitable species habitats under current and future scenarios. They also use algorithms to detect patterns in habitat loss, population decline, and human threats (poaching and logging) [91]. Thus, ML formulated wildlife corridor designs and conservation features through a systematic literature review. Machine algorithms are also used in the geolocation of current wildlife habitats and predict future suitable sites in light of global climate changes. Random forest (RF) shows great potential in species distribution modeling (SDM), predicting useful insights on the potential future habitats [127]. Mathur et al. [128] used the ensemble model, an ML-based system to assess tree (*Tecomella undulata*) species global distribution based on current and future bio-climatic (2050 and 2070) and four greenhouse (RCP2.6, 4.5, 6.0, and 8.5) scenarios, as well as soil attributes. ML-based systems also work to predict the global distribution of invasive species. Three different species distribution models (SDMs)—MaxEnt, random forest (RF), and multi-layer perception (MLP)—were used to predict the global distribution of invasive ants under current and future climates [129].

**Table 2:** AI, ML, and DL techniques application summaries in wildfire detection and management and biodiversity conservation.

Application	AI System (Model/Algorithm)	References
Tracking forest wildfires	AI models: LiDAR—UAV-LS and TLS; ground station sensor, camera, and UAVs	[57,82]
Forest-fire monitoring	AI-models: UAVs armed with infrared and visual cameras	[130]
Citrus disease detection and management	DL-based CNN with ML-based algorithms (SoftMax and RBF SVM)	[104]
Postharvest monitoring	ML-based artificial neural networks (regression trees)	[131]
Disease forecasting: rice blast prediction	ML-based conventional multiple regression (REG), back-propagation neural networks (BPNNs)	[132]
Disease assessment: leaf blast	DL-Sentinel 2: NDVI, EVI, NDMI, SAVI	[133]
Prediction of tree species distribution	ML-based models: rainforest and artificial neural networks	[134]
Above ground biomass prediction	DL models: DNNs (regression equations)	[135]
Species distribution of invasive tree species	ML-based: support vector machine (SVM)	[136]

### 4.5. Carbon Sequestration and Climate Mitigation

Accurate knowledge of forest biomass is crucial in understanding carbon stocks and the importance of forest plants in mitigating global climate change and sustainable forest management [137]. Machine learning (ML) algorithms, such as linear regression (LR), random forest (RF), and extreme gradient boosting (XGBoost), combined with remote sensing, are widely used to estimate the forest above-ground biomass. Li et al. [138] showed that the XGBosst is the best model for above-ground biomass (AGB) estimation, reducing challenges of over- and underestimating. Additional applications of ML-based models in carbon sequestration and AGB estimation are shown in Table 3.

Mapping forest structures is essential for monitoring forest resources and understanding ecological processes. Deep learning algorithms, combined with remote sensing, have improved forest structuring compared to traditional methods (Figure 4). Airborne light detection and ranging (LiDAR) characterize 3D canopy structures and estimate forest structure parameters. DL-based algorithms (Deep-RBN) combined with the fully connected network (FCN) and radial basis neural network (RBN) display strong estimation capabilities in forest structure estimations [139].

Deep learning is becoming an essential tool for predicting and modeling climate change impact due to its ability to process huge, complex data (Figure 4). It makes predictions based on environmental conditions. For instance, DL models are capable of predicting the impact of climate change on ecosystems, weather patterns, species populations, and migration (Figure 4). This gives management an advantage in mitigating future undesired impacts and improving forestry output in case of adverse climate changes [140]. Deep learning models are trained on remote sensing data, including LiDAR, multispectral, and hyperspectral imagery, to estimate forest biomass and carbon stocks. These models capture fine-grained spatial and structural details of forest canopies and understories to understand how much carbon is stored in forests, which is critical for carbon credit markets and climate change mitigation (Figure 4; Table 3). In addition, knowledge of the above-ground biomass provides a guide to sustainable harvesting practices that will encourage forest regeneration and long-term productivity. Nonetheless, optical remote sensing and LiDAR have challenges in providing full detail on spectral vegetation canopies (Figure 1). Deep learning-based workflows combined with imageries from LiDAR and Landsat 8 accurately estimate forest biomass and facilitate carbon stock assessments [141]. Satellite imageries from the Sentinel-2 multispectral instrument (MSI), Advanced Land Observing Satellite 2 (ALOS-2) L-band, and Sentinel-1 C-band SAR were also used in combination with the CNN and Keras to estimate and map the forest carbon density [142]. A study in the moist temperate forest in the Galies region of Abbottabad, Pakistan, estimated forest biomass using the Sentinel-2 remote sensing data analyzed by DLAs [143].

Artificial intelligence (AI) has a significant role in enhancing data-driven decision-making in forest management [144]. The integration of AI with digital and satellite technology has been shown to improve the monitoring, management, and conservation of forest resources [50]. These advancements facilitate the efficient surveillance and administration of forests, contributing to biodiversity preservation on a global scale.

**Table 3:** AI, ML, and DL techniques application summaries in carbon sequestration and climate mitigation.

Application	AI System (Model/Algorithm)	Reference
Prediction of CO_2_ emission	ML based-GRNN, MLPNN, RF, radial basis function neural network (RBFNN), and adaptive neuro-fuzzy inference systems (ANFISs)	[145]
Forecasting the spatiotemporal variability of soil CO_2_	ML-based artificial neural network (ANN)	[146]
Estimation of carbon sequestration	DL-based: MLP neural networks and regression analysis	[147]
Monitoring soil carbon pool	Artificial neural networks and regression models	[148]
Predicting soil carbon stocks	ML-based RF techniques and DL-based regression models	[149]
Timber harvesting; planning and predicting production and management	ML algorithms combine with GIS data	[150]
Forest harvesting scheduling	AI-based, two-phase heuristic algorithm	[151]
Conservation and distribution prediction	AL-based models with bioclimatic data	[152]
Estimation of Carbon sequestration	ML-based regression algorithms (XGBoost)	[153]
Forest fire susceptibility predictions	ML-based models—RF, multivariate adaptive regression splines (MARS), and DL models (DLNN)	[154]
Wild forest fire detection system	DL-based, attention-based convolution neural network with bidirectional long short-term memory (ACNN-BLSTM)	[155]

## 5. Conclusions and Future Perspectives

Sustainable forest management addresses concerns in forestry production and ecological balances. The application of AI technologies, deep learning, and machine learning in SFM is transforming forests, ecosystems, and forestry management. The utilization of these tools represents an evolving technology that will transform forest management practices. AI-driven algorithms provide a complete analysis of forest dynamics by analyzing data from remote sensors such as satellites, drones, and terrestrial sensors. They offer a proactive approach that promises to ensure environmental integrity and sustained economic development. The use of artificial intelligence, deep learning, and machine learning in forestry improves operational efficiency and supports sustainability by increasing resource optimization, preserving biodiversity, and managing risks effectively.

By enabling plant ecologists and SFM managers to identify and minimize early indicators of disease, pest infestations, and environmental stressors, these technologies help to avert future damage and support long-term resilience. Furthermore, improving forest product certification and traceability through deep learning and machine intelligence promotes the efficient use of forest resources and supports sustainable supply chains. As AI, DL, and ML technologies advance and provide creative answers to the intricate challenges faced by forests globally, the potential to transform sustainable forestry management will increase. By adopting these modifications and achieving an equilibrium between environmental conservation and the escalating demands on forest resources, we may enhance the protection and preservation of our forest ecosystems for future generations.

Despite the significant potential of AI, ML, and DL to enhance sustainable forestry management, various obstacles and constraints hinder their progress. The effectiveness of AI, ML, and DL integration in SFM is substantially constrained by data quality and availability. Successful algorithm training requires high-quality data, which may be challenging to obtain in forested regions. Similarly, remote sensing technology can generate low-resolution images and metadata. Another concern is that AI models, including deep learning algorithms, operate as “black boxes”, creating a challenge since forestry stakeholders may favor transparency. The requirement for transparency hinders the effective operation of AI systems. Maintaining a balance between AI-driven systems and traditional ecological knowledge may be challenging due to potential integration issues. The ethical and societal implications will affect issues such as land use rights, biodiversity conservation, and disproportionate impacts on specific groups. Societal challenges may impact AI-based systems concerning SFM. Ultimately, many stakeholders, especially from underdeveloped nations, may lack the financial or technological resources necessary for the efficient deployment of AI-based solutions. The efficient application of artificial intelligence, deep learning, and machine learning technologies will determine the future governance of sustainable forests.

These technologies will improve and refine techniques for monitoring resource utilization, assessing forest health and resources, aiding conservation initiatives, and developing sustainable supply chains. AI-powered technologies improve forestry operations through enhanced data analysis, predictive modeling, and real-time monitoring. Technological advancements will create opportunities for innovation in sustainable forestry management, thereby enhancing the equilibrium between social and economic benefits and environmental integrity.

## Figures and Tables

**Table 1:** AI, ML, and DL techniques application summaries in forest monitoring, health, mapping, and predictive analytics.

Application	AI-System (Model/Algorithm)	References
Automated forest inventory (plant tree physical features)	LiDAR: ForAINet, FOR-Instance	[80]
Tree population control	LiDAR: ALS-derived tree registry and tree maps	[81]
Tree crown metrics (crown surface and volume)	LiDAR: UAV-LS and TLS	[57,82]
Analysis for deforestation detection	ML: random forest (RF) and multilayer perceptron (MLP), Landsat Data; DL: CNN	[83]
Mapping tree species proportions	CNN (U-Net, ResU-Net, SegNet, FC-DenseNet, DeepLAbv3+)	[84,85]
Forest management (fertilizer application time and quantity)	ML methods: Randon Forest, XG Boost, Support Vector Regression, and ANN algorithm.	[86]
Tree individual crown delineation	VHR satellites, MT-EDv3 (CNN)	[87]
Estimating tropical forest carbon stock	RGB-drone imagery, CNN	[88]
Wildlife monitoring: behavior, distribution	DL: YOLOv5, Exiftool version 12.42 Grizzly-AI equipped cameras, CT-distance sampling, random encounter	[89]
Understanding insect–prey interactions, monitoring human wildlife crimes	YOLOv5	[90]
Identify habitat and predict populations	Occupancy modeling	[91,92]
Wildlife density estimation	ML algorithms (MegaDeyector, Wildlife Insights)	[93]

## Abbreviations

The following abbreviations are used in this manuscript:

SFM	Sustainable forest management
AI	Artificial intelligence
ML	Machine learning
DL	Deep learning
CNN	Convolutional neural networks
LiDAR	Light detection and ranging
UAV	Unmanned aerial vehicles
SAR	Synthetic aperture radar
NDVI	Normalized Difference Vegetation Index
DNN	Deep neural networks
ASV	Autonomous surface vehicles
AGB	Above-ground biomass
